# Metastatic Epithelioid Hemangioendothelioma of the Bone: A Case Report and Literature Review

**DOI:** 10.7759/cureus.58378

**Published:** 2024-04-16

**Authors:** Jihane Derfoufi, Mouhsine Omari, Younesse Najioui, Anass Haloui, Ouissam Al Jarroudi, Amal Bennani, Sami Aziz Brahmi, Said Afqir

**Affiliations:** 1 Medical Oncology, Faculty of Medicine and Pharmacy, Mohammed VI University Hospital, Mohammed First University, Oujda, MAR; 2 Pathology, Faculty of Medicine and Pharmacy, Mohammed VI University Hospital, Mohammed First University, Oujda, MAR; 3 Anatomopathology, Faculty of Medicine and Pharmacy, Mohammed VI University Hospital, Oujda, MAR; 4 Medical Oncology, Mohammed VI University Hospital, Oujda, MAR

**Keywords:** chemotherapy agents, molecular testing, anatomopathology diagnosis, case report, bone, metastasis, tumor, epithelioid hemangioendothelioma

## Abstract

Epithelioid hemangioendothelioma (EHE) is an extremely rare vascular tumor, which can pose a diagnostic dilemma. It affects women more than men and is mainly found in the liver, lung, and bone. To date, there are no known predisposing factors. Limited data are available on the management of EHE at metastatic stages. The only optimal treatments to prevent metastatic dissemination are surgical resection and amputation in addition to radiotherapy at early stages. The oncologist in this rare entity plays an important role in the guided and standardized management of this disease, especially for advanced stages. In this article, we report the case of a 74-year-old patient admitted with swelling on the outer aspect of the right calf associated with pain and total functional impairment of the limb. The diagnosis favored a high-risk vascular tumor resembling EHE, confirmed by bone (tibia) and soft tissue biopsy. The patient underwent staging investigations, revealing diffuse metastases to the liver, bones, and lungs. The objective of this article is to advocate for oncological intervention in this entity, particularly in the advanced stages of the disease. Despite its rarity, the advancement of clinical trials and therapeutic recommendations remains crucial for optimal treatment.

## Introduction

Epithelioid hemangioendothelioma (EHE) is an intermediate malignant vascular tumor, often multifocal [1]. It belongs to the sarcoma family, exhibiting histological characteristics that are both angiomatous and sarcomatous [2]. Its highly heterogeneous nature results in variable clinical presentations from one patient to another. It represents 1% of all vascular tumors and is locally aggressive [3]. It can be distinguished from other types of angiosarcomas with a worse prognosis by the WWTR1-CAMTA1 translocation, which is present in 90% of cases [4]. In this article, we present the clinical, radiological, and histopathological characteristics, as well as the oncological management, of a case of metastatic tibial EHE.

## Case presentation

We report the case of a 74-year-old Moroccan woman who has no known medical history, presenting with swelling of the right calf and pain, causing total functional impairment of the leg beginning in 2018. Upon clinical examination, the patient was in generally good health, with an Eastern Cooperative Oncology Group (ECOG) performance status of 2 due to pain in the right limb. Clinical examination of the affected leg revealed a large tight calf with collateral venous circulation. Palpation revealed a hard and painful region of about 20 cm. There were no palpable lymph nodes in the groin, femoral, or popliteal areas. The definitive diagnosis was difficult to establish.

**Figure 1:**
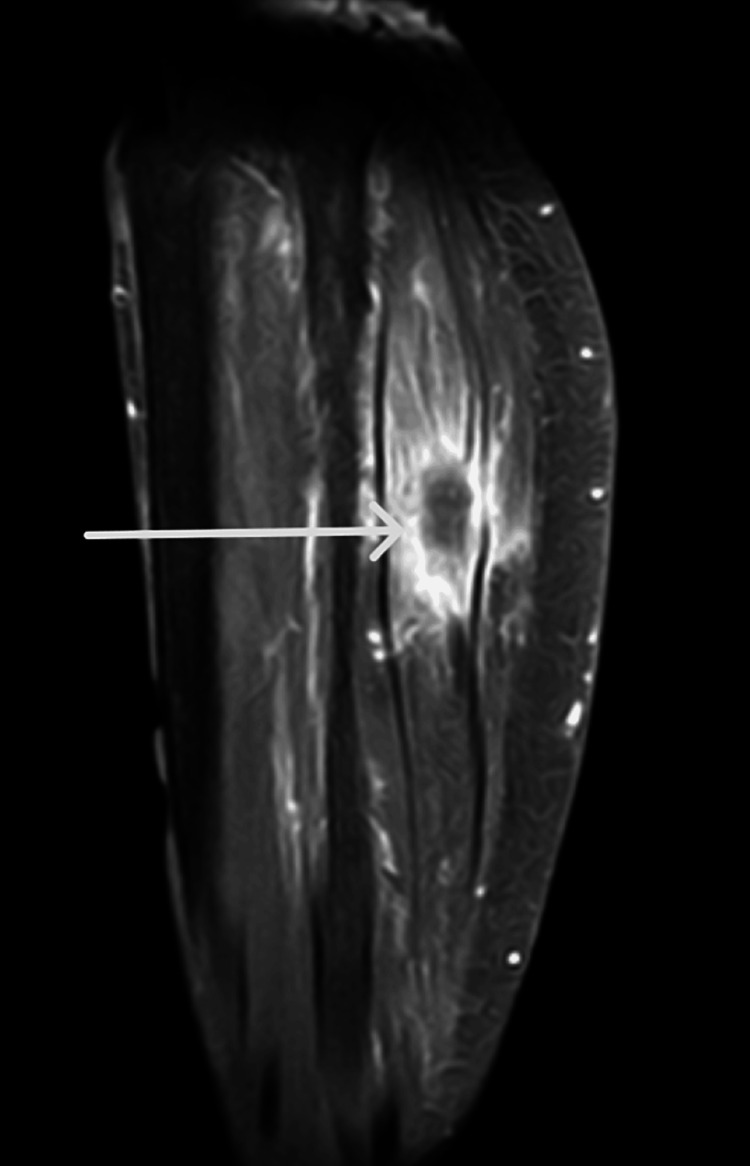
An MRI image of the right leg showing a mass (white arrow) measuring 33x33x23 mm, eroded and unevenly posterior to the fibula at the metaphyseal-diaphyseal junction

Several paraclinical tests were carried out to a final clinical diagnosis. A magnetic resonance imaging (MRI) of the right leg was performed and showed a well-defined soft tissue mass measuring 33x33x23 mm, eroded and unevenly appearing posterior to the fibula at the metaphyseal-diaphyseal junction, spanning 76 mm, with no cortical rupture or medullary change (Figure 1). A cervical, thoracic, and abdominopelvic CT scan revealed lung, liver, and bone metastases.

**Figure 2:**
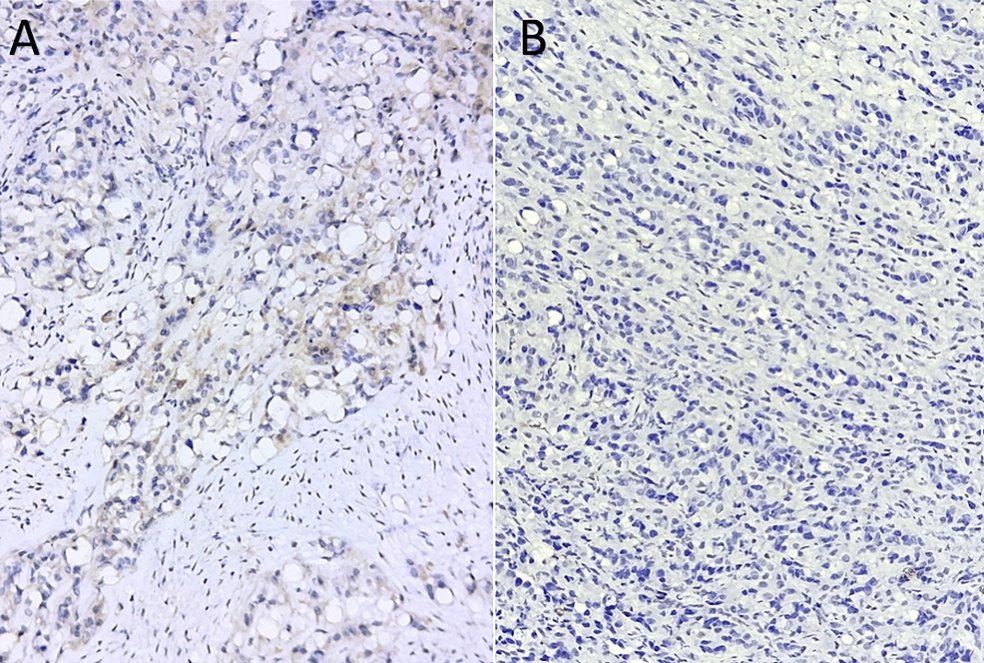
The combined expression of anti-cytokeratin and anti-EMA antibodies, indicating the characterization of epithelial origin cells A) Negative EMA expression. B) Negative cytokeratin expression

**Figure 3:**
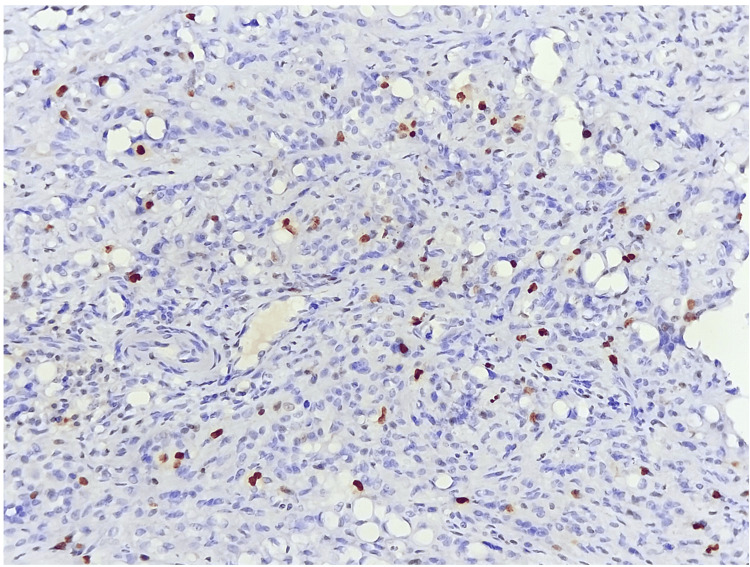
Microscopic image showing tumor proliferation estimated by Ki67 Magnification: x20

**Figure 4:**
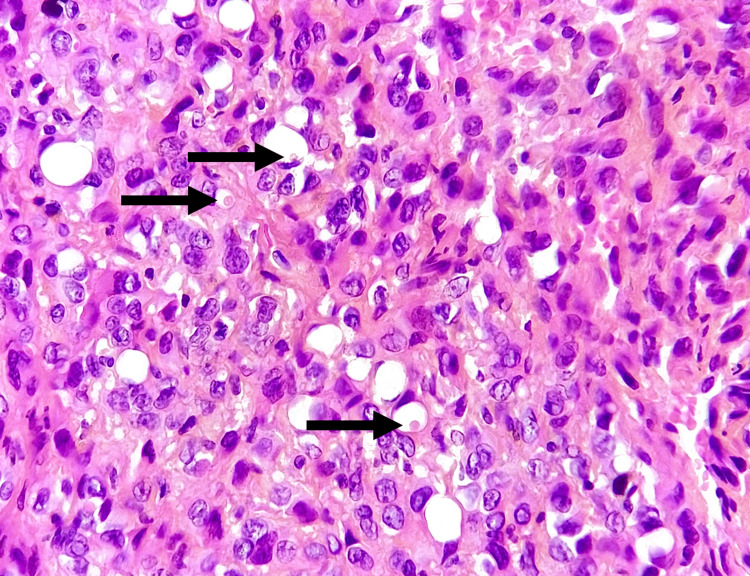
Anatomopathological image showing the vacuolar appearance of the cells (blister cells) Hematoxylin and eosin (H&E); magnification: x40

Histological and immunohistochemical tests were carried out using two samples (the bone and soft tissue adjacent to the suspicious lesion). It showed negative staining for desmin, myogenin, EMA, cytokeratin (Figure 2), PS100, CDK4, or CD34, but had strong and widespread positive staining of tumor cells for CD31, AML, and MDM2 for CD31, AML, and MDM2. Ki67 expression was estimated at 30% (Figure 3). A second test was done to evaluate CAMTA1 expression and complement fluorescence in situ hybridization (FISH). The final diagnosis favored a high-grade epithelioid hemangioendothelioma (Figure 4).

Following this argument, a pre-chemotherapy workup was conducted to evaluate the best treatment choice for the patient. Following a multidisciplinary board meeting, the patient was prescribed first-line treatment with weekly paclitaxel at a dosage of 80 mg/m^2^. The patient's recovery was characterized by successful six-cycle completion, well treatment tolerance, and good clinical response. A follow-up of 10 months was marked by tumor stability locally and on distant metastasis.

## Discussion

Epithelioid hemangioendothelioma (EHE), a very rare vascular sarcoma, was initially described by Weiss and Enzinger in 1982, as an unusual tumor with epithelioid and histiocytic features that were frequently misdiagnosed as a sarcoma [5]. EHE was first described in the lung, liver, and soft tissues and is increasingly being reported in bone through case series. Up to this date, its prevalence persists at one in every one million [6]. It is very challenging to choose the best treatment for EHE patients. No systemic therapy until now is approved. EHE is usually resistant to soft tissue sarcoma anti-tumor medications [7]. The present article describes bone EHE, which is classified as an intermediate-grade vascular tumor by the 2020 WHO but is, in fact, a malignant tumor with a high potential for metastasis in 15% of cases [8].

Epidemiologically, EHE incidence is approximately 0.38/100,000 inhabitants per year, with a frequency of < 1/100,000 persons. It occurs in middle-aged adults, with a 60% (1/4) female preponderance [9]. Lau et al. identified EHE in 206 patients, 61% female and 39% male, with an average age of 38 years; and 21% died after two years of follow-up at the end of the trial (average age 47 years). In this series, only 14% had bone sites. There was no difference in survival based on location or multifocal involvement [6]. In its osseous forms, the long bone is the most common site in more than half of the cases, but it can also be multifocal [10]. Our case was unique in the fact that the tumor was located in the tibia and had extensive axial and peripheral secondary bone lesions, as well as secondary visceral lesions.

EHE may develop anywhere in the body in a variety of forms, from a single lesion to multifocal or metastatic disseminated lesions [6]. Due to the rarity of this condition, as well as its variation of suggestive clinical symptoms, the diagnosis may be very challenging. According to a literature review by Witte et al., bone involvement can be difficult to separate from multifocal or metastatic disease, and the most prevalent clinical signs include pain, edema, congestion, and pathological fractures [11].

However, the radiological imagery observed is not specific to EHE; they can also indicate tendinous expansions to adjacent moles, malignant bone lesions with or without cortical rupture, and bone demineralization [12]. EHE usually appears as a lytic lesion with a honeycomb appearance, intermittent cortical disruption, and extension to the surrounding soft tissue. On MRI, it presents a T2 hypersignal, indicating that it is highly vascularized, while on CT, it seems homogenous. 18-FDG PET-CT is excellent for revealing the extent of bone lesions, with its strong fixation power [13,14]. Our patient underwent an MRI of the affected limb combined with CT-CTAP to determine distant tumor extension for better staging of the disease and to consider the optimal treatment.

An anatomopathological study is the basis of diagnosis, and it has progressed greatly over the history of EHE. Macroscopically, EHEs show reddish-brown, loculated masses with extensive bleeding. They emerge from the arterial wall, obliterating the vessel lumen and spreading centrifugally across the surrounding tissue [15,8]. Microscopically, it demonstrates no sign of mature vascular development, but rather a phenotype defined by the presence of intracytoplasmic vacuoles harboring erythrocytes. Epithelioid tumor cells form chain-like or string-like arrangements within a myxohyaline stroma [8,15]. Hemangioendotheliomas often have a monomorphic nuclear morphology with low-grade characteristics. Immunohistochemistry plays a major role in the diagnosis of certainty, as indicated by the presence of vascular markers (ERG, CD31) in 20% of patients but a heterogeneous expression of CD34, cytokeratin 7, or CK8, CK18, and EMA in 30% of cases [16,17].

Additional molecular testing may be performed to confirm the diagnosis. Most of the patients have a translocation t (1;3) (p36.3; q25), which results in the WWTR1-CAMTA1 gene fusion [18,19]. A less common genetic aberration has recently been identified, characterized by a translocation t(X;11) (p11; q22), which results in a YAP1-TFE3 fusion [20]. Individuals with a YAP1-TFE3 fusion are often younger than those with a WWTR1-CAMTA1 fusion [21]. Other rare fusions can also be observed. The presence of the translocation does not depend on the anatomical site or clinical behavior. The WWTR1 protein, also known as the TAZ protein, is one of the two end effectors of the Hippo pathway. WWTR1 and YAP play a pro-oncogenic role. Oncogenic transformation is the outcome of the Hippo pathway's loss of regulation caused by the fusion of WWTR1 and CAMTA1 [22]. An expert anatomopathological opinion is needed to establish the diagnosis of EHE.

The treatments of metastatic EHE are not well elucidated due to a lack of phase III studies and no therapeutic standard. Several small case series reported various therapies employed, which we tried to explain in this paper in order to point out the treatments that are effective for this entity. Because bone forms of EHE are extremely rare, therapy is chosen based on the various hepatic forms, or on the investigator's selection from the few accessible case reports of bone forms.

Our case is unique in the way that it focuses on a multifocal metastatic bone type that cannot be treated surgically. Systemic treatment is still the only option currently accessible. The treatment was chosen based on a small series of patients who received paclitaxel as first-line therapy, after a multidisciplinary board meeting.

In a 1985 study, five patients with metastatic hepatic EHE who had chemotherapy followed by radiation had survival rates of up to 96 months with stable metastases [23]. No single chemotherapy regimen has been found to be 100% successful, and the chemotherapy methods employed are based on a succession of EHEs involving many locations. In numerous documented case series, anthracyclines were the most commonly used first-line chemotherapeutic agents [24-26]. A series of 29 patients with multicentric or localized bone EHE was published in 1986, with a follow-up of 33 months after surgery or adjuvant radiotherapy. They concluded that the ideal treatment is surgery whenever possible [27]. Since the series published by Royal Marsden from 1999 to 2012, paclitaxel has been utilized in 28% of patients, indicating a satisfactory clinical analgesic response and performance status improvement for a median treatment period of three months [28]. Additionally, in a retrospective series of 73 patients by the World Sarcoma Network, 11 patients treated with paclitaxel had a PFS of 2.9 months and an overall survival of 18.6 months. Various therapies such as 5FU, platinum, interferon (IFN), and tyrosine kinase inhibitors have been studied in a variety of series, but with limited objective results: the majority of patients progressed [29]. Bevacizumab was tested in an open-label phase II study on 32 patients with locally advanced or metastatic angiosarcomas or hemangioendothelioma, and it is a well-tolerated option for treatment [30].

A recently published phase II trial evaluating trametinib in patients with locally advanced or metastatic EHE. Trametinib reduced pain and extended progression-free survival by six months, without reaching its primary endpoint of overall survival [31]. A second ongoing research was studying a possible treatment for angiosarcoma and hemangioendothelioma using eribulin for metastatic patients; if successful, it may be a new potential therapy (NCT03331250).

## Conclusions

EHE of the bone is an extremely rare kind of vascular sarcoma. The diagnosis of confirmation is anatomopathological, with immunohistochemical and molecular biological tests. Given the minimal number of case series with metastatic bone EHE, as well as the lack of clinical studies determining the optimum modality for managing this entity, the decision to treat with paclitaxel was made through case series. Despite the rarity of metastatic bone EHE, a standardized systemic therapy at these phases is required for better patient management.
